# The Effect of Odor Valence on Facial Attractiveness Judgment: A Preliminary Experiment

**DOI:** 10.3390/brainsci12050665

**Published:** 2022-05-19

**Authors:** Guo Feng, Jiawei Lei

**Affiliations:** 1 Psychological Research and Counseling Center, Southwest Jiaotong University, Chengdu 611756, China; leijiawei2017@163.com; 2 Institute of Applied Psychology, Southwest Jiaotong University, Chengdu 611756, China

**Keywords:** odor valence, facial attraction, cross-modal integration, olfaction

## Abstract

The role of social odors on human social interactions, including face evaluation, has been widely indicated. However, for nonsocial odors, there has not been a consistent conclusion. Therefore, this study aimed to verify the effect of suprathreshold nonsocial odors on facial attractiveness judgment when the visual input is ambiguous. We designed a 3 (odor valence: neutral, pleasant, and unpleasant) × 7 (continuous levels of morphed fuzziness of attractiveness: 37.5% to 62.5%) within-subject experiment. A total of 30 participants (18 females) completed the whole experiment simultaneously for three consecutive days. The results showed that faces presented with pleasant and neutral odors were judged as significantly more attractive than those with unpleasant odors. The intervention effect of odor valence on facial attractiveness differed by fuzzy attractiveness levels. Results also suggested that male faces were perceived as more attractive than female faces no matter the odor conditions. The results of this study provide evidence to support the cross-modal emotion integration effect of olfaction and vision. Follow-up studies need to be conducted to reveal the underlying mechanism of odor valence on visual fact attractive judgment.

## 1. Introduction

“What is good is beautiful” [1]. Facial attractiveness is a trait that induces a positive and pleasant mood and drives others to a certain level of willingness to approach [2], which leads individuals with high facial attractiveness to have a significant advantage in social activities [3]. Moreover, facial attractiveness is associated with health status, genetic quality, and reproductive capability [2,4]. It is generally believed that facial attractiveness is evaluated mainly through visual cues [5,6], while olfactory cues can also modulate the judgment of facial attractiveness [7]. Numerous studies have indicated that social chemosignals (e.g., androstadienone, estratetraenol, etc.) can significantly enhance perceived attractiveness [8,9]. However, there is no consistent conclusion on whether nonsocial odors (such as perfume) could affect the judgment of facial attractiveness.

The impact of the olfactory sense on humans’ life is great and multifaceted, and humans actually can distinguish subtle differences in odors [10]. In addition, olfactory cues exert significant cross-modal influence on perception [11], emotion [12], and even attraction judgments [11]. Notably, these results are sometimes inconsistent or contradictory. For example, odor valence strongly influences subjects’ likeability rating of neutral expression faces when the olfactory stimuli are delivered unconsciously [7], while this effect disappears when participants become aware of the odors [7]. Nevertheless, other studies have shown that suprathreshold odor can also interfere with the evaluation of faces [13,14]. By presenting different valence odors and neutral expression faces simultaneously, another study suggested that the faces that appeared with unpleasant odors were perceived as less attractive [15]. Moreover, when faces were presented with an unpleasant odor, the effect on an individual’s perception of facial attractiveness was greater than when faces were presented with a pleasant odor or a neutral baseline [13,15,16].

An increasing number of researchers have begun to explain the influence of olfactory cues on facial evaluation from the perspective of cross-modal effects [17]. The integrated processing assumption, which makes participants believe that they perceive sensory stimuli (both olfactory and visual) from the same object or event, argued that the face should be presented at the same time as the odors [17]. Research has found that when odors are presented before faces (e.g., spraying the room with perfume in advance), participants are less likely to associate the odor with the presented face [15,17]. furthermore, olfaction is particularly linked with human emotion [18], and valence is the most important attribute of odors. Thus, the valence of odor and the evoked emotional results may impact the pleasantness processing from the visual modality. An MRI study showed that when faces were presented with fragrance, increased activation was observed in the orbitofrontal cortex and the medial part of the ventral striatum [15]. In contrast, activation increased in the insula and amygdala, which is known to be associated with the processing of negative stimuli [19], when the same faces were paired with an unpleasant odor.

It is also worth noting that the judgment of attractiveness would appear to be influenced to a greater extent compared with other social appraisals [11]. As for the perception of facial attractiveness, on the one hand, faces with average features are more attractive than faces with extreme features based on the averaging hypothesis [5,20]. On the other hand, real sensory and emotional responses cannot be accurately represented by extreme dichotomy. In addition, cross-modal integration of sensory information helps humans understand the external world more accurately and quickly [11,14]. This integration follows an inverse effect; that is, when one kind of information input is more ambiguous, the other kind of sensory input has a stronger moderating effect on that modality [21,22]. Hence, to investigate the odor valence effect on face evaluation, it is necessary to weaken the visual face cues of attractiveness that participants can perceive as much as possible. This may partially explain why previous studies on the influence of odor on facial attractiveness have not been able to reach a consensus [7,13].

Another possible reason may be traditional Likert rating used in most facial attractiveness studies is not sensitive enough to reveal the olfactory biasing effect. Generally, rating response is subjective and time-consuming. Studies indicated that facial attractiveness could be assessed rapidly and modulates brain processes as early as 150 ms after a face is encountered [23,24]. Furthermore, almost no extreme rating values would be given by participants through using the rating paradigm, and this reduces the validity and reliability of the evaluation [25]. Correspondingly, Best–Worst Score (BWS), a forced-choice method as a potential alternative to the Likert scale, efficiently measures participants’ facial attractiveness [26].

Based on the above evidence, to investigate whether odor valence can bias visual judgment of facial attractiveness, especially when the visual input is ambiguous, we morphed the faces with high and low attractiveness and only fuzzy attractiveness faces in the middle sequence, which are difficult to distinguish, were selected as the visual materials. In addition, face fuzziness would weaken visual cues and enhance the olfactory cues’ effect on facial attractiveness judgment if the effect existed. Moreover, we adopted a dichotomous forced-choice method to record participants’ rapid response to ambiguous faces that may reflect their real evaluation. Therefore, the present study hypothesizes that (1) pleasant odors would make participants perceive fuzzy attractiveness faces as more attractive, while unpleasant odors would result in the opposite; (2) the effect of odor valence is different for images with different fuzziness of attractiveness; specifically, the fuzzier the face is, the stronger the effect of odor intervention is.

## 2. Method

### 2.1. Participants

Based on the calculation method in Cohen (1977) [27] and the medium effect size (f = 0.25), as well as the expected efficacy value (power = 0.80) of relevant early study [21], G * Power 3.1 software was used to calculate the planned sample size of more than 28 participants. Therefore, 30 undergraduates (12 males and 18 females; M_age_ = 20.17, SD_age_ = 1.42; no gender difference, χ^2^ = 1.2, *p* > 0.05) from Southwest Jiaotong University were recruited through posters, and they all completed the whole study. They reported having a normal or corrected-to-normal vision, a normal sense of smell, and no respiratory allergy or upper respiratory infection at the time of testing. They were all right-handed, nonsmokers, and gave informed consent to participate in procedures approved by the Institutional Review Board of SWJTU. During experimental days, participants were blind to the study purposes. After the whole procedure, they would be informed of the real purpose of the experiment and be paid 50 RMB.

### 2.2. Materials and Measures

#### 2.2.1. Olfactory Stimuli

The olfactory stimuli were presented in identical felt-tip pens [28], each filled with 1 mL liquid. They consisted of a neutral odor (diluted in water), a pleasant odor (essential oil with an orange-like smell, 10 mL 0.34 fl.oz, Puressentiel Inc., Paris, France, FRA), and an unpleasant odor (5% concentration valeric acid, diluted in propylene glycol). The selection of different valence odors was motivated by those used in previous studies [7,15,29]. Additionally, the odorants were judged as different valence (M_pleasant_ = 6.35, M_unpleasant_ = 1.78, *t* = 10.51, *p* < 0.05) and matched in intensity (M_pleasant_ = 6.86, M_unpleasant_ = 7.38, *t* = −1.39, *p* > 0.05) by an independent sample (*n* = 20, half females and half males; M_age_ = 21.66, SD_age_ = 1.82, no gender difference, $\chi^{2}$ = 0.43, *p* > 0.05). For odor presentation, the lid of the cap of the pen was removed, and the pen tip was placed approximately 2 cm in front of the two nostrils. Participants were instructed to sample the olfactory stimuli by inhaling through the marker’s tip and exhaling through the mouth throughout the experiment.

#### 2.2.2. Visual Stimuli

**Figure 1:**
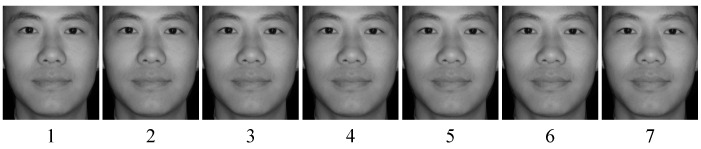
Levels of applied morphing. The illustrations are examples of morphed faces. We selected seven morphs, ranging from somewhat high attractiveness to somewhat low attractiveness. These images were morphed to be attractive from 62.5% to 37.5%; level 4 was the most ambiguous (50% attractive).

The visual stimuli were 56 moderately attractive and ambiguous morphed facial images generated through a sequence of operations. Firstly, we selected 100 pictures of neutral expression male faces and 100 pictures of neutral expression female faces from the Chinese Affective Face Picture System (CAFPS) [30]. Then, the appearance attractiveness of these faces was rated in an independent group (*n* = 18, 10 females and 8 males; M_age_ = 22.94, SD_age_ = 1.63, no gender difference, $\chi^{2}$ = 0.22, *p* > 0.05) on a visual analog scale from 1 (very low) to 100 (very high). Next, we ranked the rating scores of those pictures from high to low and chose 8 highest-rated pictures and 8 lowest-rated pictures (both the highest group and lowest group were composed of 4 male faces and 4 female faces) as visual pictures for further steps. We also used a sample *t*-test to verify that there are significant differences in attractiveness between highly attractive faces and lesser attractive faces (*p* < 0.05) and no significant rating difference between female faces and male faces (*p* > 0.05). Finally, by randomly pairing highly attractive and lesser attractive same-gender faces and by using 4.17% increments from high to low attractiveness, we generated 8 unique series containing 200 morphs and a list of 25 graded attractive morphs (Abroaoft Fantamorph 5.1, https://www.fantamorph.com/, accessed on 15 December 2021). To choose the ambiguous morphs to be used in this study, we selected 7 images in the middle of the series (Figure 1); thus, a total of 7 × 8 = 56 images (28 females) were adopted as the visual stimuli.

All visual stimuli were presented on a 13.3-inch monitor, with Windows 10 computer system, i5 processor, and 1920 × 1080 pixels screen resolution. They were placed in the center of the monitor with a white background and a visual angle of 6.57° in height and 10.29° in width. All facial images were frontal views with the same grayscale and brightness, 260 × 300 pixels, and a dark gray background. The hair and ears in each image had been removed (Figure 1).

#### 2.2.3. Questionnaire

The questionnaire consisted of two parts. The first part was demographic information including gender, age, and vision, in addition to other questions that may impact olfactory function (e.g., medical history, smoking status, rhinitis condition, contraceptives, or hormonal medication). The other part was the Positive and Negative Affect Scale (PANAS) to assess participants’ emotional changes during the experiment. The PANAS consists of 20 items and two subscales: positive (e.g., interested) and negative (e.g., upset) emotions [31,32]. It uses a five-point scale from 1 (not at all) to 5 (extremely strong) to evaluate participants’ emotions at the time of the experiment. The positive emotion subscale was subtracted from the negative emotion subscale to obtain a total score. The higher the total score, the more positive the participant’s emotion.

### 2.3. Procedure

The present study had a 3 (odor valence: pleasant, unpleasant, and neutral) × 7 (continuous levels of morphed fuzziness of attractiveness: 37.5% to 62.5%) within-participant factorial design. It was conducted in an independent lab around the same time for three consecutive days. The order of odors presented every day was random and balanced.

**Figure 2:**
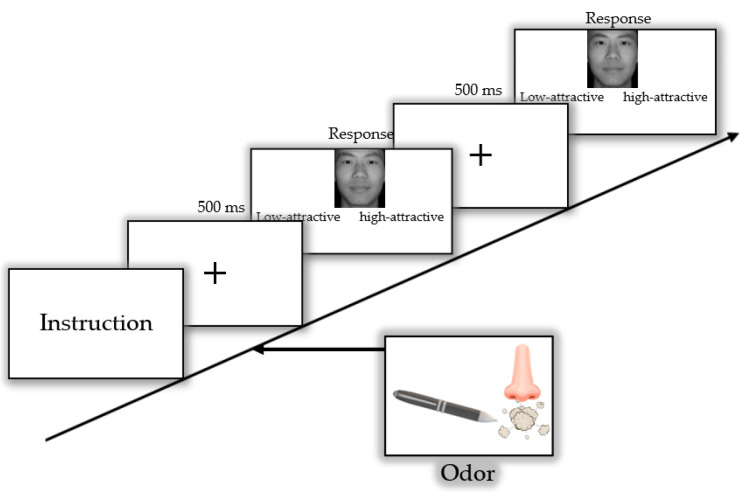
Selection procedure of three (neutral, pleasant, unpleasant) conditions. During the task, participants were asked to indicate whether each face was a high- or low-attractive one. First, they saw a fixation for 500 ms, and then an image was presented. The next trial began immediately after the participant responded.

On each day, participants were first required to complete PANAS before and after the experiment. Then, they were asked to rate the familiarity, intensity, and pleasantness of odors as soon as presented, using a scale of 1 (not familiar, low intensity, not pleasant) to 9 (very familiar, high intense, very pleasant) points. In the third step, participants performed 560 trials of a forced attractiveness judgment task. Each generated picture was repeated 10 times, and all these stimuli were randomly ordered. All these trials were divided into 5 blocks. In each block, participants were instructed to hold the pen tip approximately 2 cm in front of their nose and sample the olfactory stimuli by inhaling through the marker’s tip and exhaling through the mouth till the end of this block. There was a 2 min break between blocks. In each trial of the task, a fixation “+” first appeared in the center of the screen for 500 ms, and then a face image was presented until participants had used the left (low-attractive) and right (high-attractive) arrows of the keyboard to choose whether the face was highly attractive or not. Participants were asked to respond as accurately and as quickly as possible. Then, the next trial began immediately after the participant responded (Figure 2).

### 2.4. Data Analysis

Behavioral data were collected using E-Prime 3.0 software (PST Inc., Sharpsburg, PA, USA). We collected the responses of participants and calculated the proportion of “high-attractive” judgment responses of the task repeated ten times. All data are continuous and obey normal distribution. Odor-rating scores in both pleasant and unpleasant conditions were analyzed using an independent sample *t*-test. Data from the experimental task was analyzed using 3 × 7 repeated-measures ANOVA to assess the difference in odor valence and continuous levels of morphed fuzziness of attractiveness. PANAS scores’ difference was analyzed using one-way ANOVA. The SPSS 26.0 (IBM Inc., Armonk, NY, USA) statistical software package was used to conduct one-way ANOVA, independent sample *t*-test, and Spearman correlation. *p*-value < 0.05 was considered statistically significant. All data were used in two-tailed tests. All figures and receiver operating characteristic curve (ROC) were drafted with Origin 2021 (OriginLab Inc., Northampton, MA, USA).

## 3. Results

### 3.1. Odor-Rating Results

After excluding extreme scores (two standard deviations), the difference between pleasantness, intensity, and familiarity of pleasant and unpleasant odors was tested by an independent sample *t*-test. The results showed the pleasant odor had a significant higher pleasantness rating score (M = 6.40) than the unpleasant odor (M = 1.90), *t* = 11.89, *p* < 0.001; no intensity difference was found between these two odors (*p* > 0.05); familiarity rating score of the pleasant odor was slightly higher than unpleasant odor (*p* = 0.09) (Table 1).

In addition, we compared the different impacts of pleasant and unpleasant odors on participants’ moods; we subtracted the pre-task PANAS score from the post-task score and tested the changing value. The results indicated that the total scores of PANAS decreased significantly more in the unpleasant odor condition (M = −7.37) than in the neutral condition (M = −2.23, *t* = 3.62, *p* < 0.01) and pleasant odor condition (M = −1.93, *t* = −3.85, *p* < 0.001). No significant difference was found between neutral and pleasant odor conditions.

### 3.2. Odor Valence and Morphing Levels Influence Facial Attractiveness Judgment

**Figure 3:**
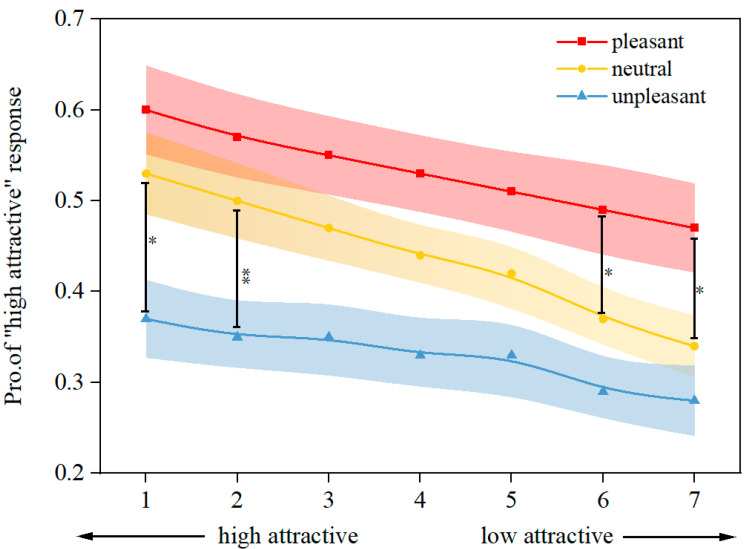
Effects of odor valence and morphing levels on the proportion of responses choosing “high-attractive” faces. As a function of morphing level and odor condition, the proportions of faces classified as highly attractive are shown. The variations were calculated. Significant differences between conditions are indicated using one-way ANOVA (a black dash line between the dots of levels 1, 2, 6, and 7). * *p* < 0.05, ** *p* < 0.01.

A repeated-measures ANOVA revealed a significant main effect of odor valence on the chosen proportion of high attractiveness (*F*_(2, 28)_ = 15.16, $\eta_{p}^{2}$ = 0.52, *p* < 0.001). Faces presented with unpleasant odor were judged as less attractive than neutral condition (*p* < 0.001) and pleasant condition (*p* < 0.001). Faces presented with pleasant odor were judged as more attractive than neutral condition (*p* < 0.001). There was a main effect of morphing levels on proportion of choosing “high-attractive” option (*F* = 6.88, $\eta_{p}^{2}$ = 0.63, *p* < 0.001). level 1 was significantly judged as more attractive than level 6 (*p* < 0.01) and level 7 (*p* < 0.001); level 2 was more attractive than level 7 (*p* < 0.01). Moreover, a significant interaction effect of odor valence and morphing levels on facial attractive judgment was found (*F*_(12,18)_ = 4.97, $\eta_{p}^{2}$.= 77, *p* < 0.001). Further, one-way ANOVA indicated that odor valence has a significant influence on the attractiveness judgment at all facial morphing levels. At each level, the proportion of responses choosing a “high-attractive” face in pleasant odor conditions was significantly higher than that in unpleasant odor conditions (*ps* < 0.05). Significant results were also found between unpleasant and neutral conditions in levels 1 and 2 (*ps* < 0.05). Faces presented with pleasant odors were more judged as “high-attractive” faces than those presented in neutral conditions in levels 6 and 7 (*ps* < 0.05) (Figure 3).

**Figure 4:**
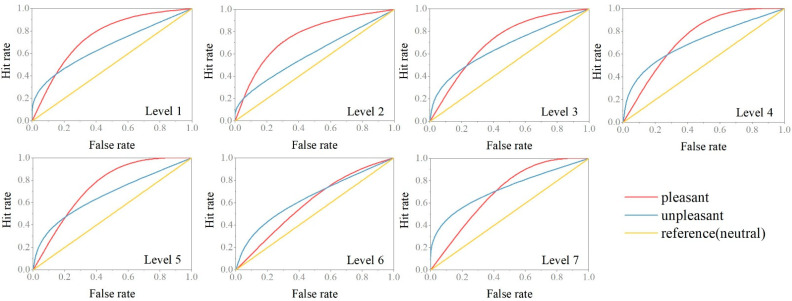
Receiver operating curve. ROC was used to evaluate the accuracy of a binary classifier. A false rate represents a false-positive rate, while a hit rate represents a true-positive rate.

To further evaluate how participants’ judgments were influenced, we set values larger than the ideal ratio (morphing percentage, e.g., level 1 should have a 62.5% probability of being judged as highly attractive) to 1 and values less than the ideal ratio to 0. Additionally, we set the data in the neutral condition as control values and set positive and negative conditions as test values. The hit-and-false rate calculations were employed to produce the receiver operating characteristic (ROC) curve (Figure 4). The area under the ROC curve (AUC) was calculated using the Roccurve toolkit (Origin 2021) and collected for further statistical analysis.

Compared with the neutral condition, both pleasant and unpleasant odors increased participants’ perception of facial attractiveness (AUC > 0.5). AUC value is equivalent to the probability that a randomly chosen positive example is ranked higher than a randomly chosen negative example [33]. The larger the value of AUC in the positive condition, the more likely subjects were to rate the face as highly attractive. In pleasant odor conditions, participants were more likely to judge the faces as highly attractive, and this proportion showed a significantly decreasing trend as the level increased (r = −0.78, *p* < 0.05). In unpleasant odor conditions, participants were more likely to judge faces as lesser attractive, and this tendency tended to gradually rise as the level increased (r = 0.41, *p* > 0.05).

### 3.3. Odor Valence and Gender Affect Facial Attractiveness Judgment

**Figure 5:**
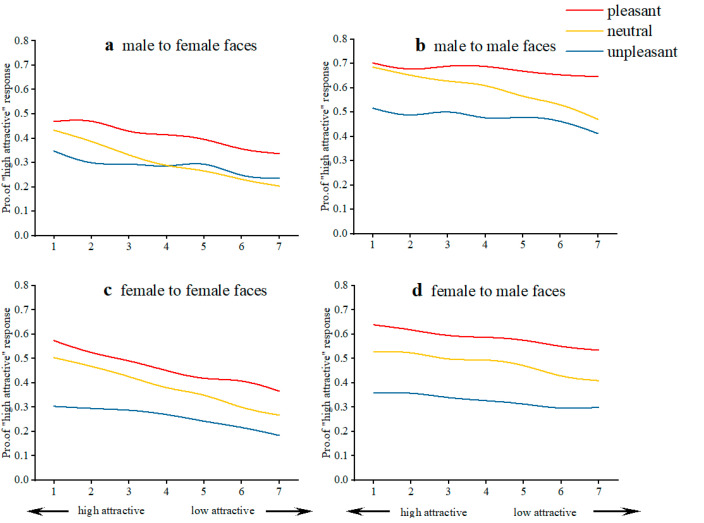
Effect of gender and odor valence on facial attractiveness. Gender consistency induced attractiveness biases along the valence dimension: male participants viewing female faces (**a**); male participants viewing male faces (**b**); female participants viewing female faces (**c**); female participants viewing male faces (**d**).

Considering that the attractiveness judgment of faces may be related to the gender of facial images and the gender of the participants themselves, we further analyzed the effect of gender (participant gender and face image gender) on facial attractive judgments in combination with odor valence. The results showed that all participants classified male faces as higher proportion chosen of highly-attractive (M_pro._ = 0.52) than those of female faces (M_pro._ = 0.35, *t* = 7.24, *p* < 0.001). For male participants, male faces (M_pro._ = 0.58) were judged more attractive than female faces (M_pro._ = 0.33, *t* = 9.05, *p* < 0.001). Even when male faces were presented at the same time with unpleasant odors (M_pro._ = 0.48), they were rated more attractive than female faces that were presented with pleasant odors (M_pro._ = 0.41, *t* = 2.54, *p* < 0.05). For female participants, male faces (M_pro._ = 0.46) were also judged as more attractive than female faces (M_pro._ = 0.37, *t* = 2.78, *p* < 0.05). Specifically, female participants judged male faces (M_pro._ = 0.48, 0.59, 0.33) more attractive than same-gender faces (M_pro._ = 0.39, 0.46, 0.26) in all neutral, pleasant, and unpleasant odor conditions (*ps* < 0.01) (Figure 5).

## 4. Discussion

In this study, we investigated the effect of odor valence on facial attractiveness through a forced judgment paradigm. Participants were required to evaluate a series of moderately attractive and ambiguous, morphed facial images in different odor conditions. The results showed that, compared with unpleasant odor conditions, faces were judged as more attractive when presented at the same time with neutral and pleasant odors. Thus, hypothesis 1 was proved. Both positive and negative odors can increase participants’ perceptual ability. In addition, the intervention effect of odor valence on facial attractiveness differed with different levels of morphing fuzziness of attractiveness, especially at levels 1, 2, 6, and 7, which partially proved hypothesis 2. Meanwhile, an unexpected result showed that all participants generally perceived male faces as having higher facial attractiveness, irrespective of the odor conditions under which they were presented.

The present study provided evidence to support that facial attractiveness can be modulated by nonsocial olfactory information when the visual stimuli task is ambiguous and difficult to evaluate. This result supports the theory that information integration across sensory modules follows an inverse effect [22] and is consistent with previous studies [21,34]. Specifically, it means that when visual information is ambiguous, the odor can dominate visual perception. For example, Zhou et al. (2010) conducted a binocular rivalry paradigm study to verify this effect. In their study, presenting the picture of a whiteboard pen to the left eye and the picture of a rose to the right eye, the subject would perceive the whiteboard pen seen by the left eye and the rose seen by the right eye for a while. Then, when he smelt the rose, he observed the picture of the rose for a longer time, and vice versa. This shows that the weight of pictures in the visual processing system can be regulated by olfactory input [35]. In our study, the seven facial images that morphed most fuzzily were used as visual stimuli. Therefore, it was reasonable to observe a significant effect of odor valence on the judgment of facial attractiveness even if we used nonsocial odors.

Furthermore, the results also showed that the judgment of facial attractiveness was influenced differently by the simultaneous presentation of different valence odors for different fuzziness of attractiveness. Generally, the most ambiguous face should be maximally affected by odor based on the above inverse effect [34]. However, in our study, the strongest effects were found at levels 1, 2, 6, and 7, not at levels 3, 4, and 5, which featured more fuzziness of attractiveness. These results may serve as further verification that the cross-modal effects of odor valence on the judgment of facial attractiveness are nonlinear. Specifically, when the difficulty of visual discrimination exceeds a certain degree, the regulatory effect of odor is not enough to produce significant behavioral changes. The specific neural mechanisms need to be further explored in the future.

The current study also found that both pleasant and unpleasant valence odors could enhance participants’ sensitivity to facial attractiveness. From a broader perspective, the processing of olfactory and visual stimuli is regulated by attention [36]. On the one hand, the integration of olfactory and visual information can enhance the saliency of the corresponding object and attract attention. Research has shown that when participants smelled odors, they were presented with different pictures to the left and right of their field of vision at the same time. Even if the subjects’ eyes did not move, their attention would be attracted by the pictures consistent with the smell. In more complex scenes, such as when looking at more than a dozen pictures of all kinds and with the same color, odors can still help participants to find the same one faster [37]. On the other hand, the pleasantness of the odor can selectively divert human attention spans [36] and guide attention to displayed features that are valence congruent with the scent [38]. This, to a certain extent, can explain the enhancement effect of odor valences on attraction judgment when odors and faces were presented simultaneously in this study. An unexpected result in this study is that the proportion of male faces evaluated to be highly attractive was higher than that of female faces in all odor conditions. Studies have shown that men pay more attention only to highly attractive faces, while women have a higher sensitivity to all facial stimuli and have a high attentional bias to both highly attractive and lesser attractive faces [39]. As in the process of mate selection, women pay more attention to men’s social resources and social status than to men’s faces [39], and this may make women less concerned about the attractiveness of male faces. Therefore, due to individuals’ limited cognitive resources, men choose to attribute more value to facial features, while women choose to pay more attention to same-sex competitors and hold stricter evaluation criteria [39,40].

There are several limitations in the present study. Firstly, the participant sample is relatively smaller than in other studies of attractiveness. More participants should be recruited to repeat the experimental results. Secondly, to increase the odor valence effect on facial attractiveness, we weakened the visual cues as much as possible, which may affect the ecological validity of this experiment to a certain extent. Finally, the pleasant odor was more familiar than the unpleasant odor, and this may indicate that the effect found in this study was partially due to the different levels of familiarity. Follow-up studies are needed to further control the familiarity of olfactory stimuli. In addition, impression management is an important part of our social activity. Exploring other social impressions influenced by odors is of great interest and practicability.

## 5. Conclusions

The present preliminary study provided evidence to support the cross-modal emotion integration effect of olfaction and vision. It was suggested that both pleasant and unpleasant odors can significantly modulate an individual’s perception of facial attractiveness when seeing faces with neutral expressions, and the effect intensity may depend on the morphed fuzziness of facial attraction. The underlying mechanism of odor valence affecting the visual perception of facial attraction needs to be further explored in a follow-up study.

## Figures and Tables

**Table 1:** Ratings of odor pleasantness, intensity, and familiarity.

	Pleasant Odor Mean (SD)	Unpleasant Odor Mean (SD)	*t*
Familiarity	6.32(1.07)	5.57(1.75)	1.71
Intensity	6.60(1.19)	7.01(1.33)	−1.33
Pleasantness	6.40(1.38)	1.90(1.14)	11.89 ***

Note: *** *p* < 0.001.

## Data Availability

All data and models used during the study are available from the corresponding author by request.
